# The Impact of Sequence of Chemotherapy and EGFR-TKI Treatment on Different *EGFR* Mutation Lung Adenocarcinoma

**DOI:** 10.1155/2015/948267

**Published:** 2015-11-02

**Authors:** Fu-Tsai Chung, Ming-Yun Ho, Yueh-Fu Fang, Meng-Heng Hshieh, Tsai-Yu Wang, Chih-Hsi Kuo, Hao-Cheng Chen, Chun-Hwa Wang, Shu-Min Lin, Chih-Teng Yu, Horng-Chyuan Lin, Han-Pin Kuo

**Affiliations:** ^1^Department of Thoracic Medicine, Saint Paul's Hospital, Taoyuan 330, Taiwan; ^2^Department of Thoracic Medicine, Chang Gung Memorial Hospital at Linkou, Chang Gung University, College of Medicine, Taipei 10507, Taiwan; ^3^Department of Internal Medicine, Saint Paul's Hospital, Taoyuan 330, Taiwan; ^4^Department of Internal Medicine, Chang Gung Memorial Hospital at Linkou, Chang Gung University, College of Medicine, Taipei 10507, Taiwan

## Abstract

*Objectives*. Chemotherapy as first-/second-line treatment in different epidermal growth factor receptor (*EGFR*) mutation lung adenocarcinoma remains controversial. *Methods*. Consecutive patients were collected between 2009 and 2012. Patients were divided into two groups (1st-line chemotherapy: *n* = 56 and 2nd-line chemotherapy: *n* = 55). Their outcomes profiles were analyzed. *Results*. The overall survival (OS) of all patients (390 versus 662 days, *p* < 0.0001), as well as both progression-free survival (PFS, 151 versus 252 days, *p *= 0.0001) and OS (308 versus 704 days, *p* = 0.0001) of patients with *L858R* mutation (*n* = 63), who received 2nd-line chemotherapy, was significantly poor. By univariate and multivariate analysis, 2nd-line chemotherapy, and *L858R* mutation were significantly related to poor PFS and OS. *Conclusion*. In advanced lung adenocarcinoma, *L858R* mutation and 2nd-line chemotherapy caused a poor outcome. It is a consideration to choice of 1st-line chemotherapy in these subjects. A prospective design is warranted to confirm this finding.

## 1. Introduction

The major forms of lung cancer are non-small-cell lung cancer (NSCLC) and small-cell lung cancer. NSCLC is divided into three subtypes: squamous-cell carcinoma, adenocarcinoma, and large-cell lung cancer. Most of lung cancer in nonsmoker group is adenocarcinoma [1]. It is often diagnosed at advanced stage and has poor prognosis. The systemic therapy is appropriated for advanced lung cancer. The choice of systemic therapy includes chemotherapy or the agents to specific molecular pathway. The option of treatment depends upon tumor histology, stage, molecular characteristics, performance status, and underlying diseases [1].

The mutant epidermal growth factor receptor (EGFR) is a specific molecular pathway in NSCLC [2]. The mutant* EGFR* in adenocarcinoma lung cancer is approximately 10% in United States and 30~50% in Asia [3]. The tyrosine kinase inhibitors (TKIs) such as gefitinib and erlotinib are target to EGFR. Those agents are oral active form and effective for advanced NSCLC [4, 5]. They can preferentially affect malignant cells but less to affect normal cells.

In recent years, the clinical benefits of TKIs were discovered in* EGFR* mutation, nonsmokers, women, adenocarcinoma, and Asian ethnicity patients [6, 7]. The 1st-line TKIs in mutant* EGFR* NSCLC had better progression-free survival (PFS) than chemotherapy. However, there was no significant finding in overall survival (OS) [7, 8]. It may relate to further systemic therapy after disease progression.

The patients with advanced NSCLC can receive 1st-line TKIs or chemotherapy followed by 2nd-line chemotherapy or TKIs as alternative treatment. The choice of 1st-line therapy between chemotherapy or TKIs is controversial. Despite the better PFS in TKIs therapy, the OS was still not significant in many trials [8, 9]. There are less available data about the sequence of TKIs or chemotherapy in NSCLC. The different sequence of systemic therapy may have different efficiency.

In this study, the mutant* EGFR* advanced lung adenocarcinoma patients were collected from the cohort database. The efficiency between 1st chemotherapy/2nd TKIs and 1st TKIs/2nd chemotherapy group was compared. From the cohort database survey of mutant* EGFR* lung adenocarcinoma, we conducted this study to evaluate the response and survival in different sequence of chemotherapy/TKIs among all and different* EGFR* mutation subgroup patients.

## 2. Methods

### 2.1. Data Collection

The patients were included from Linkou Chang Gung Memorial Hospital and Saint Paul's Hospital at Taoyuan, Taiwan. The inclusion criteria were advanced lung adenocarcinoma with mutant* EGFR* patients, who received TKIs or platinum-based doublet chemotherapy as the 1st-line treatment. The data was collected between 2009 and 2012. Patients received two kinds of therapies (EGFR-TKIs and chemotherapy) at different sequence were included. They were divided into two groups, 1st-line TKIs/2nd-line chemotherapy and 1st-line chemotherapy/2nd-line TKIs. The general data including age, smoking status, gender, performance status (PS), and* EGFR* mutation status before treatment were surveyed. The response after treatment was evaluated according to RECIST (Response Evaluation Criteria In Solid Tumors) criteria. The PFS and OS were investigated in two groups. The institutional review board of the institute approved the study.

### 2.2. Response

The tumor response to chemotherapy was assessed with computed tomography every two to three cycles, but tumor response was also evaluated at any time if signs of tumor progression were noted. Objective responses of tumor to chemotherapy were based on the Response Evaluation Criteria in Solid Tumors (RECIST) [10]. Partial response (PR) was defined as at least a 30% decrease in the sum of diameters of target lesions, using the baseline sum diameters as a reference. Progressive disease (PD) was defined as at least a 20% increase in the sum of diameters of target lesions, using the smallest sum of the study as a reference (this includes the baseline sum if it is the smallest of the study). In addition to the relative increase of 20%, the sum must also demonstrate an absolute increase of at least 5 mm. The appearance of one or more new lesions was also considered progression. Stable disease (SD) was defined as neither sufficient shrinkage to qualify for PR nor a sufficient increase to qualify for PD, with respect to the smallest sum of diameters while in the study.

### 2.3. Survival Evaluation

PFS and OS were estimated in two different sequences of TKIs/chemotherapy group. The OS from each arm of treatment was calculated from the start date of the treatment until death or until the last follow-up visit. OS took all deaths into account. PFS included the time from the first cycle of chemotherapy or the date of TKIs treatment to documented progression until death from any cause or until the date of the last follow-up visit for patients who were still alive.

### 2.4. Determination of* EGFR* Mutations

The method to detect mutation was reported in our previous report [11]. Briefly, tissue sections from formalin fixed and paraffin embedded specimens were examined for* EGFR* mutations using the* EGFR* PCR Kit (QIAGEN Inc., Valencia, CA) according to the manufacturer's instruction. By combining two technologies, ARMS and Scorpions, this kit enabled the detection of the most prevalent somatic mutations in the EGFR gene, which were common in human cancers. Surgically resected tissue samples containing tumor cell proportion of at least 10% or biopsy samples of at least 30% were determined as adequate. Tumor cell enrichment was achieved using Laser capture microdissection for those with <60% tumor cell proportion while DNA was extracted by the QIAamp DNA FFPE Tissue Kit (QIAGEN Inc., Valencia, CA). QPCR was performed in a Rotor-Gene Q real-time PCR cycler (QIAGEN Inc., Valencia, CA) using the protocol provided by the* EGFR* PCR Kit. Samples with repeated negative control reactions were interpreted as inadequate or poor quality DNA.

### 2.5. Statistical Analyses

The baseline characteristics of patients, including age, sex, smoking history, and PS were analyzed by *t*-test or Chi-square test with a contingency table. OS and PFS were both analyzed in this study. Survival curves were estimated by the Kaplan-Meier method, whereas the log-rank test was used to compare the patient survival times per group. OS was calculated from the start date of treatment until patient death. PFS included the time from the start of treatment to documented progression until death from any cause. The Cox proportional model was fit to estimate the hazard ratio (HR) for the calculated risk factors. Univariate and multivariate analyses were performed to identify independent factors contributing to PFS and OS in all patients, and in those with* EGFR* (*L858R*) mutation to control for possible confounding factors. Univariate analyses were primarily used to select variables based on *p* < ⁡0.05. These variables were then entered into a multinominal logistic regression analysis to identify the net effects of each individual factor. The median PFS and median OS were used as dependent variables in analyzing contributing factors to PFS and OS, respectively. Odds ratios (OR) and their 95% confidence intervals (CI) were computed with a logistic regression model to clarify the impact of several potentially independent prognostic factors. Statistical analysis was performed using SPSS software (version 10.0, SPSS, Chicago, IL, USA) and Prism 5 for Windows (version 5.01, Graphpad Software, Inc., San Diego, CA, USA), and statistical significance was considered at *p* < 0.05.

## 3. Results

### 3.1. Patients

During the study period, 468 patients with advanced non-small-cell lung cancer were surveyed. We excluded the patients who were nonadenocarcinoma, without* EGFR* mutation and unknown* EGFR* status. There were 55 patients who received 1st TKIs/2nd chemotherapy and 56 patients who received the 1st chemotherapy/2nd TKIs. The general data in two groups was presented in Table 1. There was no difference in two groups including age, gender, smoking status, performance status, and* EGFR* mutation status.

### 3.2. Outcomes Analyses

#### 3.2.1. Response

*Response to 1st-Line Treatment*. The disease control rate (81.8% versus 89.3%, *p* = 0.26) and response rate (54.5% versus 41.7%, *p* = 0.16) to 1st-line treatment of two groups were not significantly different. Nevertheless, stable disease rate (47.6% versus 27.3%, *p* = 0.02) of 1st-line TKI/2nd-line chemotherapy was better than that of another group (Table 2).

#### 3.2.2. Patients Receiving 2nd-Line Treatment

Of 55 patients who received 1st-line TKI/2nd-line chemotherapy, only 27 patients received 2nd-line treatment; meanwhile, among 56 patients who received 1st-line chemotherapy/2nd-line TKIs, most patients (*n* = 52) could receive 2nd-line treatment. The rate of patients who received 2nd-line treatment was significantly different (49.1% versus 92.9%, *p* < 0.0001) between 1st-line TKI/2nd-line chemotherapy group and 1st-line chemotherapy/2nd-line TKIs group.

#### 3.2.3. Response to 2nd-Line Treatment

The response rate (11.1% versus 25%, *p* = 0.15) and stable disease rate (33.3% versus 46.2%, *p* = 0.27) to 2nd-line treatment of two groups were not significantly different. Nevertheless, disease control rate (44.4% versus 71.2%, *p* = 0.02) to 2nd-line treatment of 1st-line TKI/2nd-line chemotherapy group was worse than that of another group (Table 2).

#### 3.2.4. Survival

**Figure 1:**
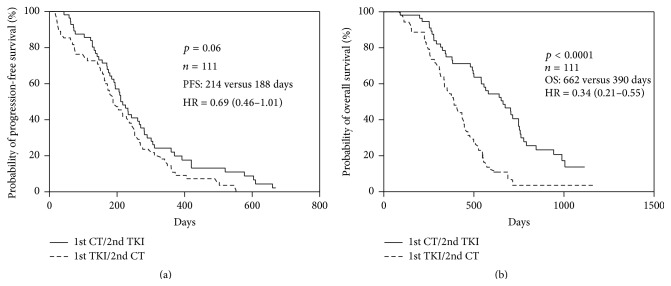
Kaplan-Meier curve of (a) progression-free survival (PFS) and (b) overall survival (OS) of 1st-line treatment for all patients (*n* = 111) receiving 1st-line chemotherapy/2nd-line TKIs and 1st-line TKIs/2nd-line chemotherapy.

Among all included patients (*n* = 111), the PFS of patients who received 1st-line chemotherapy/2nd-line TKIs was similar to that of another group (median PSF: 214 versus 188 days, hazard ratio (HR): 0.69; 95% confidence interval (CI): 0.46–1.01; *p* = 0.06, Figure 1(a)). The OS was significantly different between two groups (1st-line chemotherapy/2nd-line TKIs versus 1st-line TKIs/2nd-line chemotherapy: 662 versus 390 days, HR: 0.34; 95% CI: 0.21–0.55; *p* < 0.0001, Figure 1(b)).

**Figure 2:**
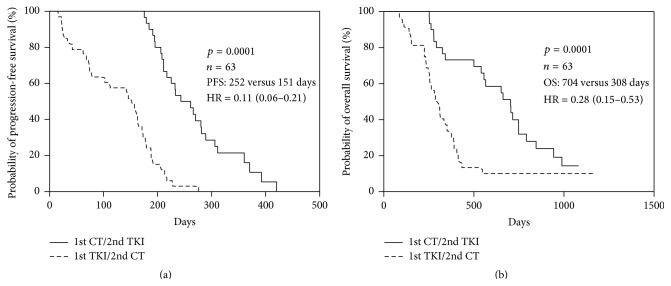
Kaplan-Meier curve of (a) progression-free survival (PFS) and (b) overall survival (OS) of 1st-line treatment for* L858R* mutation patients (*n* = 63) receiving 1st-line chemotherapy/2nd-line TKIs and 1st-line TKIs/2nd-line chemotherapy.

Among patients with* L858R* mutation (*n* = 63), both PFS and OS of patients were significantly different between two groups (Figure 2(a), median PSF: 252 versus 151 days, hazard ratio (HR): 0.11; 95% confidence interval (CI): 0.06–0.21; *p* = 0.0001; Figure 2(b), 1st-line chemotherapy/2nd-line TKIs versus 1st-line TKIs/2nd-line chemotherapy: 704 versus 308 days, HR: 0.28; 95% CI: 0.15–0.53; *p* = 0.0001, Figure 1(b)).

### 3.3. Univariate Analysis of Clinical Factors for PFS and OS

#### 3.3.1. Among all Patients

The median PFS and OS were 198 and 497 days, respectively. Among the clinical factors examined, poor PS, positive smoking history, and 2nd-line chemotherapy treatment and* EGFR* mutation (*L858R* versus* 19del*) were significantly related to poor PFS and poor OS (Table 2). 2nd-line chemotherapy was associated with poor PFS (HR: 2.1; 95% CI: 1.6–4.9; *p* = 0.01) and poor OS (HR: 2.3; 95% CI: 1.4–4.7; *p* = 0.01) when analyzed with a log-rank test. The Kaplan-Meier survival curves for PFS and OS, according to 1st-line or 2nd-line chemotherapy treatment (Figures 1 and 2), revealed a clear trend toward poor survival outcomes for those patients with 2nd-line chemotherapy (Table 3).

#### 3.3.2. Among Patients with Known* EGFR* (*L858R*) Mutation

To take* EGFR* mutation into consideration, the results were also analyzed in the subset of patients with known* EGFR* (*L858R*) mutation (*n* = 63). Among the clinical factors examined, poor PS, positive smoking history, and 2nd-line chemotherapy significantly correlated with poor PFS and poor OS (Table 3).

### 3.4. Multivariate Analysis of Factors for PFS and OS

To determine independent factors for PFS and OS, multivariate analysis was performed and included significant factors determined in univariate analysis (Table 4). 2nd-line chemotherapy was significant for PFS (OR: 1.7; 95% CI: 1.57–3.89; *p* = 0.03) and OS (OR: 1.8; 95% CI: 1.4–3.9; *p* = 0.01). Similarly,* EGFR* mutation with* L858R* was also significant for PFS (OR: 2.2; 95% CI: 1.7–4.5; *p* = 0.02) and OS (OR: 2.6; 95% CI: 1.2–5.6; *p* = 0.01). These data indicate that L858R and 2nd-line chemotherapy were significant factors for PFS and OS, independent of other confounding factors.

## 4. Discussion

As IPASS data revealed NSCLC patients with mutant* EGFR* had a high response rate to 1st-line TKI treatment, 30% patients with lung adenocarcinoma and EGFR mutation had no response to 1st-line EGFR-TKI treatment [8]. Recently, TORCH study reported that first-line erlotinib followed at progression by cisplatin-gemcitabine was significantly inferior in terms of overall survival compared with the standard sequence of first-line chemotherapy followed by erlotinib in unselected patients with advanced NSCLC [12]. Therefore, the 1st-line treatment to patients with mutant* EGFR* advanced lung adenocarcinoma is an important issue whether chemotherapy or EGFR-TKIs selected. Our study demonstrates that the 1st-line chemotherapy followed by EGFR-TKIs in patients with* EGFR* mutation advanced lung adenocarcinoma had a better OS than that of the 1st-line TKIs followed by 2nd-line chemotherapy in these patients (median: 662 versus 390 days, *p* < 0.0001; Figure 1(b) and Table 2). Most of these patients (52/56, 92.9%) could shift to 2nd-line TKIs when disease progressed after 1st-line chemotherapy, meanwhile, less patients (27/55, 49.1%) could receive 2nd-line chemotherapy when disease progressed after 1st-line TKIs treatment. The PFS to 2nd-line treatment in 1st chemotherapy/2nd TKIs group is significantly better than that of 1st TKIs/2nd chemotherapy group (median: 199 versus 85 days, *p* < 0.0001; Table 2); similar trend was found in PFS to 1st-line treatment despite being without statistical significance (median: 214 versus 188 days, *p* = 0.06; Figure 1(a) and Table 2).

All of patients in this study had mutant* EGFR* advanced lung adenocarcinoma and both groups had similar performance status. The choice of initial therapy is influenced by financial problem and the toxicity of chemotherapy. There has been no comparative data between first- and second-line* EGFR*-TKIs in patients with activating* EGFR* mutations so far. However, tumor response rates to second-line* EGFR*-TKIs are inconsistent. It may be due to the effect of first-line chemotherapy on the abundance of tumor cells with activating* EGFR* mutations.

Despite IPASS or West Japan Oncology Group trial revealing that the PFS is better in 1st-line TKIs than chemotherapy, the OS is not significantly different. The possible reason may be caused by the further systemic therapy which may affect the final survival outcomes. Therefore, we also conducted a prospectively randomized study to investigate the differential influence of prior chemotherapy on the efficacy of erlotinib in patients with advanced non-small-cell lung cancer (ClinicalTrials.gov Identifier: NCT01204307). The prospective study had been completed and will be reported in near future. Nevertheless, from current study, we supported that PFS could be a guide of treatment response evaluation to further treatment after TKIs or chemotherapy as the 1st-line treatment. Some studies also showed prolonged PFS with 2nd-line TKIs after 1st-line chemotherapy [13–15]. The benefit of TKIs in mutant* EGFR* NSCLC patients is confirmed in 1st-line or 2nd-line therapy. It is reasonable to switch another treatment when initial treatment (with TKI or chemotherapy) was failure. TKIs had adverse effect of skin rash, diarrhea, pulmonary toxicity, and hepatic toxicity [16]. Most of adverse effect is mild and tolerable in old age patients [17]. When 1st-line TKIs are prescribed in advanced NSCLC, the disease progression is noted due to secondary mutation of* EGFR* and* MET* oncogene amplification [18, 19]. The further systemic therapy will be given. There is no randomized clinical trial to compare different regimens of 2nd-line chemotherapy after 1st-line TKIs treatment.

Prior study reported that, after 1st-line platinum-based doublet chemotherapy, the efficiency of 2nd-line treatment between TKIs and chemotherapy after chemotherapy was no different [20]. Another report also presented that the TKIs can be a choice of maintenance therapy after four cycles of chemotherapy [21]. TKIs and chemotherapy both are still effective as 2nd-line therapy. In clinical practice, the crossover treatment is usually noted when disease progresses. In TORCH trial, the standard treatment (1st chemotherapy/2nd TKIs) is better than experimental treatment (1st TKIs/2nd chemotherapy) in unselected NSCLC patients. However, there is small portion of mutant* EGFR* patients (*N* = 39) in TORCH study. The total PFS is longer in standard treatment but there is not statistically significant (HR: 1.71, 95% CI: 0.82 to 3.60) in mutant* EFGR* NSCLC patients [12].

In mutant* EGFR* advanced lung adenocarcinoma patients, the efficiency of TKIs has been demonstrated in other trials [8, 9, 12]. However, the other systemic therapy contributes the survival outcome and makes the similar OS of patients who received 1st line TKIs or 1st line chemotherapy [8]. The timing of chemotherapy may be important. The 2nd-line chemotherapy after TKIs may not be as potent as 1st-line chemotherapy. The major cause is the nature course of disease progression and the toxicity of chemotherapy. It includes malnutrition, decondition, tumor enlargement, metastasis, and performance deterioration. All of the above reasons will affect the interval, dose, and regimen of chemotherapy. They are associated with the response of chemotherapy. Another reason is the consideration of adverse effect and patient's willingness. Many patients could not complete course of 2nd-line chemotherapy after 1st-line TKIs treatment. Only 67.5% patients continue 2nd-line chemotherapy in TKIs group and the other group is 94.6% patients receiving 2nd-line TKIs after chemotherapy [22]. Our data also revealed similar result; furthermore, we found most patients receiving 1st TKI treatment usually suffering rapid progression and worse performance status when disease progressed. The poor performance status of these patients caused them no chances to receive further chemotherapy! The survival duration will be shorted if there is no further systemic therapy because of the rapid progression and poor performance status. In addition, in those patients who could receive 2nd-line treatment, better disease control rate and PFS to 2nd-line treatment in 1st chemotherapy/2nd TKIs group were also found in our data (Table 2). These reasons may explain that the 1st chemotherapy/2nd TKIs is better than 1st TKIs/2nd chemotherapy group.

In Taiwan, the National Health Insurance provided TKIs use in mutant* EGFR* NSCLC. However, the duration of detection of mutation status of* EGFR* and application of TKIs usually costs two weeks at least. The initial therapy may be delayed because of those reasons. The 1st-line chemotherapy can reduce the timing of initial treatment. The further decision of TKIs use can be done after four- to six-cycle chemotherapy according to American Society of Clinical Oncology Clinical Practice Guideline [23].

In addition, possible and important reasons to explain our data are clinical trials of Lux-Lung 3 and 6 by Yang et al. [24]. The results of the trials revealed that the outcomes of patients who harbored 19Del and L858R were different from 1st-line* EGFR*-TKI treatment. In subgroup analysis of the trials, patients with L858R may not benefit from 1st-line* EGFR*-TKI; reversely, these subgroup patients may had better outcome from 1st-line chemotherapy! Similarly, in our current study, most patients harbored* EGFR* mutation with* L858R* in both groups (up to 55–58%); meanwhile, less patients were with* 19Del* (38–41%). This distribution of different* EGFR* mutation may explain why the outcomes of our patients were poor in those patients who received chemotherapy as the second-line treatment.

*Limitation*. First, it was not a prospective study; the character of the current study may cause bias. Second, the numbers of the current study were small so the bias should be considered. As presentation of Table 1, we listed the progression sites from each 1st-line treatment and PS at the beginning of 2nd-line treatment. Patients in 1st-line TKI group had more central nervous system (CNS) metastasis and poor performance in trend but statistical insignificance was found. The relatively small numbers in this study may explain the condition. However, patients with 1st-line TKI revealed the trend of worse performance at the beginning of 2nd-line treatment from the study. Similarly, small numbers may cause statistical insignificance. Despite the baseline condition and underlying disease of patients being balanced in Table 1, this study was not a random control trial. Some potential or unknown factors may be unbalanced due to the nonrandomized setting. Therefore, the occurrence of a skewed population cannot be excluded. The results of this study need to be confirmed by large-scale prospective randomized studies.

## 5. Conclusion

According to our study, the better overall survival is found in 1st chemotherapy/2nd TKIs group, especially among those patients with* L858R* mutation. The effect of chemotherapy may be better as an initial therapy of NSCLC, especially in mutant* EGFR* with* L858R* group. It is a consideration for the choice of systemic therapy sequence. In addition, similar to Lux-Lung 3 and 6 trials, our data also provided a different but important consideration of 1st-line chemotherapy treatment in patients with L858R EGFR mutation. The further perspective study shall be evaluated in the future. Although it is not appropriate to make such a conclusion with this study, a prospective randomized study is warranted to confirm these findings. Finally, the possible larger response rate seen in this Asian population and in others reported in the literature is worthy of further study both in terms of biomarkers and possible differences in pharmacogenomics.

## Figures and Tables

**Table 1:** Demography.

Characteristic	1st TKIs/2nd chemotherapy	1st chemotherapy/2nd TKIs	*p* value
	(*n* = 55)	(*n* = 56)	
Age, yr, median (IQR)^*∗*^	59 (52–69)	58 (52–64)	0.15
Female, *n* (%)	30 (54.5%)	27 (48.2%)	0.5
Smoking, *n* (%)	25 (45.5%)	17 (30.4%)	0.1
Performance status at beginning of 1st-line treatment			
0-1	42 (76.4%)	47 (83.9%)	0.32
2	9 (16.4%)	8 (14.3%)	0.76
>2	4 (7.3%)	1 (1.8%)	0.16
*EGFR* mutation status			
*19Del*	21 (38.1%)	23 (41.0%)	0.74
*L858R*	32 (58.2%)	31 (55.4%)	
Others	2 (3.7%)	2 (3.6%)	
Progression site from each 1st-line treatment			
Primary lung	17 (30.9%)	19 (33.9%)	0.73
Lung to lung metastasis	10 (18.2%)	9 (16.1%)	0.77
Bone	6 (10.9%)	9 (16.1%)	0.43
Pleural effusion	6 (10.9%)	7 (12.4%)	0.79
CNS	9 (16.4%)	5 (8.9%)	0.52
Liver	3 (5.5%)	2 (3.6%)	0.63
Adrenal gland	2 (3.6%)	3 (5.4%)	0.66
Others	2 (3.6%)	2 (3.6%)	0.99
Performance status at beginning of 2nd line treatment			
0-1	29 (52.7%)	38 (67.9%)	0.10
2	21 (38.2%)	15 (26.8%)	0.20
>2	5 (9.1%)	3 (5.3%)	0.45

^*∗*^IQR: interquartile range; CNS: central nervous system.

**Table 2:** Outcomes of stage IV, mutant *EGFR* lung adenocarcinoma patients who received 2nd-line versus 1st-line chemotherapy.

Outcome	1st TKIs/2nd chemotherapy	1st chemotherapy/2nd TKIs	*p* value
	*N* = 55	*N* = 56	
Response to 1st-line treatment, *n* (%)			
Disease control (PR + SD)	45 (81.8%)	50 (89.3%)	0.26
PR	30 (54.5%)	23 (41.7%)	0.16
SD	15 (27.3%)	27 (47.6%)	0.02
PD	10 (18.2%)	6 (10.7%)	0.26
Patients received 2nd-line treatment	27 (49.1%)	52 (92.9%)	<0.0001

	*N* = 27	*N* = 52	

Response to 2nd-line treatment, *n* (%)			
Disease control (PR + SD)	12 (44.4%)	37 (71.2%)	0.02
PR	3 (11.1%)	13 (25%)	0.15
SD	9 (33.3%)	24 (46.2%)	0.27
PD	15 (55.6%)	15 (28.8%)	0.02
Survival time, days, median (IQR)^*∗*^			
OS	390 (254–499)	662 (342–773)	<0.0001
PFS to 1st-line treatment	188 (102–276)	214 (142–310)	0.06
PFS to 2nd-line treatment	85 (59–148)	199 (67–354)	<0.0001

^*∗*^IQR: interquartile range; PR; partial response; SD: stable disease; PD: progressive disease; OS: overall survival time; PFS: progression-free survival.

**Table 3:** Clinical molecular factors for progression-free and overall survival in all patients (*n* = 111) and patients with known *L858R* mutation (*n* = 63) by univariate analysis.

	All cases (*n* = 111)				Patients with known *L858R* mutation (*n* = 63)			
	Progression-free survival		Overall survival		Progression-free survival		Overall survival	
	HR (95% CI)	*p* value	HR (95% CI)	*p* value	HR (95% CI)	*p* value	HR (95% CI)	*p* value
Age (y)	1.2 (0.7–1.8)	0.56	1.4 (0.8–2.2)	0.32	1.2 (0.6–2.5)	0.7	1.1 (0.5–2.2)	0.8
Gender	1.4 (0.9–2.2)	0.14	1.3 (0.8–2.3)	0.22	1.5 (0.8–2.7)	0.4	1.6 (0.8–2.9)	0.4
PS >2	2.9 (1.3–6.1)	0.02	6.8 (2.9–11.5)	<0.001	5.4 (1.6–12.5)	0.01	12.1 (6.7–15)	<0.001
Smoking	2.3 (1.3–3.7)	0.03	2.3 (1.2–3.8)	0.01	3.7 (1.5–7.5)	0.01	3.3 (1.1–7.2)	0.001
2nd-line C/T	2.1 (1.6–4.9)	0.01	2.3 (1.4–4.7)	0.01	3.9 (1.7–6.8)	0.001	4.3 (2.0–8.7)	0.001
*EGFR* mutation (*L858R*)	2.4 (1.3–5.6)	0.02	2.1 (1.4–4.9)	0.01	—	—	—	—

*EGFR*: epidermal growth factor receptor gene; HR: hazard ratio; CI: confidence interval; PS: performance status; C/T: chemotherapy; *p* values were obtained by log-rank test.

**Table 4:** Final multivariate model for progression-free survival and overall survival.

	Progression-free survival		Overall survival	
	OR (95% CI)	*p* value	OR (95% CI)	*p* value
Poor PS: >2	1.5 (0.5–4.2)	0.58	1.9 (0.81–3.82)	0.49
Smoking	1.3 (0.6–4.7)	0.49	1.4 (0.7–2.2)	0.31
2nd-line C/T	1.7 (1.51–3.89)	0.03	1.8 (1.4–3.91)	0.01
*EGFR* mutation (*L858R* to *19Del*)	2.2 (1.7–4.5)	0.02	2.6 (1.2–5.6)	0.01

OR: odds ratio; CI: confidence interval; PS: performance status; C/T: chemotherapy; *EGFR*: epidermal growth factor receptor gene.

*p* values were obtained by multinominal logistic regression analysis.
